# Exploratory Analysis of Genetic Variants in BDNF, GABA Receptors, and Dopaminergic Pathways with Alcohol Use Disorder in a Spanish Cohort

**DOI:** 10.3390/ijms27125376

**Published:** 2026-06-15

**Authors:** Maura Rojas-Pirela, Sandra Patricia Gómez Lesmes, Daniel Salete-Granado, Hernán Llorente, María-Ángeles Pérez Nieto, Ignacio Novo-Veleiro, Clara Cieza-Borrella, Isabel Pastor, Javier Fernández-Mateos, Sandra M. Inés Revuelta, Antonio-Javier Chamorro, Francisco-Javier Laso, Rogelio González-Sarmiento, Miguel Marcos

**Affiliations:** 1 Alcoholism Unit, Department of Internal Medicine, University Hospital of Salamanca, 37007 Salamanca, Spain; 2 Institute of Biomedical Research of Salamanca (IBSAL), 37007 Salamanca, Spaingonzalez@usal.es (R.G.-S.); 3 Department of Medicine, University of Salamanca, 37007 Salamanca, Spain; 4 Department of Internal Medicine, Complejo Asistencial de Zamora, Salud Castilla y León, 49022 Zamora, Spain; 5 Home Hospitalization and Palliative Care Service, Santiago de Compostela and Barbanza Health Area, 15706 Santiago de Compostela, Spain; ignacio.novo.veleiro@usc.es; 6 Department of Molecular and Biomedical Sciences, School of Health and Medical Sciences, City St George’s, University of London, London SW17 0RE, UK

**Keywords:** GABRA1, GABRA2, GABRA6, DRD2, ANKK1, BDNF, polymorphisms, genetic susceptibility

## Abstract

Alcohol use disorder (AUD) is influenced by genetic factors that affect key neurobiological systems, including dopaminergic and GABAergic pathways, which regulate neurobehavioral functions and are modulated by brain-derived neurotrophic factor (BDNF). Variations in these genes contribute to individual vulnerability to AUD. In this study, we investigated single-nucleotide polymorphisms (SNPs) and haplotypic associations in *GABRA1*, *GABRA2*, and *GABRA6*, along with the dopaminergic pathway genes *DRD2*/*ANKK1* and *BDNF*, in a Spanish cohort. Peripheral blood-derived genomic DNA was genotyped, and haplotype analyses were conducted. Individual SNPs in *GABRA1*, *GABRA2*, BDNF, and *DRD2*/*ANKK1* showed no significant associations with AUD. In *GABRA6*, the rs3219151 T allele was more frequent in AUD patients than in controls (57.9% vs. 49.3%; *p* = 0.03), while the C allele appeared to show a potential protective association. In addition, the GAC haplotype of *GABRA6* (rs2197414, rs1992647, rs3219151) was less frequent in AUD than in controls (0.071 vs. 0.122) and showed a protective association (OR = 0.58; 95% CI = 0.34–0.99; *p* = 0.045). Our findings provide exploratory evidence suggesting that specific genetic variants and haplotypes may contribute to AUD susceptibility and support the relevance of multigenic and haplotypic approaches for exploring the neurobiological mechanisms underlying AUD.

## 1. Introduction

Alcohol use disorder (AUD) is a common mental disorder associated with substantial disease burden. In 2019, alcohol consumption was responsible for approximately 2.6 million deaths globally [1]. Europe remains one of the regions with the highest alcohol consumption levels, and Spain is among the European countries with elevated per capita alcohol intake, ranking sixth in per capita intake [2]. Thus, reducing harmful alcohol consumption remains a major public health priority in Europe, particularly in high-consumption countries such as Spain.

The development of AUD results from the interaction of genetic (which account for up to 50% of the risk, according to some studies), neurobiological, and environmental factors [3,4]. Consequently, numerous studies have investigated the genetic mechanisms underlying AUD vulnerability and clinical heterogeneity. Recent genome-wide association studies (GWAS) and polygenic analyses have shown that AUD has a highly polygenic architecture involving numerous loci of small effect across multiple neurobiological pathways [5,6,7]. In addition, polygenic risk and cross-disorder genomic studies have identified shared variants related to reward processing, synaptic signaling, and addiction-related mechanisms [8].

Apart from genome-wide association studies, our research group and others have identified significant associations between genetic variants involved in neurobiological and immune-regulatory pathways and susceptibility to AUD [9,10,11]. However, important gaps remain; despite the identification of multiple genetic variants and neurobiological pathways associated with AUD, these factors collectively explain only part of its heritability and phenotypic variability, suggesting that additional mechanisms likely contribute to its pathogenesis [12,13]. Moreover, candidate-gene association studies have shown inconsistent replication across populations, partly due to small sample sizes, phenotypic heterogeneity, ancestry-related differences, and limited statistical power [14].

Nevertheless, current evidence does not fully explain interindividual risk or clinical heterogeneity, and data are limited for specific loci, ancestries, and underrepresented regions, which highlights the need for additional studies in specific populations.

In this setting, the consumption of alcohol has been linked to alterations in several neurobiological systems, particularly those involved in dopaminergic and GABAergic neurotransmission [15,16,17], which interact closely to regulate brain function and behavior in the context of alcohol addiction [12]. Among the GABAergic system, γ-aminobutyric acid subtype A receptors (GABA_A_Rs) genes, *GABRA1*, *GABRA2*, and *GABRA6*, have been linked to susceptibility to alcohol dependence [18]. Polymorphisms in these genes, such as rs279858 (*GABRA2*) and rs3219151 (*GABRA6*), are associated with susceptibility to AUD, tolerance, and neurobiological adaptation to prolonged alcohol exposure [9]. Regarding the dopaminergic system, the *DRD2* gene (dopamine receptor D2) and its associated region, the *ANKK1* (ankyrin repeat and kinase domain containing 1 gene), are among the most intensively and extensively studied genes in alcoholism and other behavioral disorders [19,20]. Notably, the *DRD2*/*ANKK1* Taq1A polymorphism (rs1800497) has been associated with reduced dopamine D2 receptor binding and altered reward processing, potentially promoting alcohol-seeking behavior [21].

Another gene of interest is *BDNF* (brain-derived neurotrophic factor), a key regulator of neuroplasticity that modulates both glutamatergic and GABAergic neurotransmission. Chronic alcohol consumption can lead to a decrease in BDNF levels in the brain and blood, potentially contributing to neuroadaptive changes involved in AUD [22]. The *BDNF* rs6265 (Val66Met) polymorphism can affect the function and expression of *BDNF* [23], as well as increase vulnerability to AUD [17,23]. Importantly, these polymorphisms have been investigated in various patient cohorts from different population groups; however, to our knowledge, studies conducted in European populations remain scarce [24,25,26,27,28,29].

Polymorphisms in *BDNF*, *GABRA1/2/6*, and *DRD2*/*ANKK1* are also linked to somatic consequences of chronic alcohol intoxication (especially liver cirrhosis) as well as depressive symptoms during abstinence [23,30,31,32]. However, these associations show inconsistency across studies, which may stem from demographic and environmental factors [33,34,35,36], as well as methodological limitations including small sample sizes, limited replication, and the isolated analysis of individual polymorphisms. These limitations support the need for integrative multigene and haplotype-based approaches that simultaneously evaluate multiple genetic variants in functionally interrelated genes, such as *BDNF*, *GABRA2*, and *DRD2*, all of which are involved in synaptic plasticity and neuroregulation of the reward system. The inclusion of haplotype analyses, in addition to the study of individual polymorphisms, could reveal relevant genetic interactions that may remain undetected in single-marker analyses. Taken together, these observations support the need for exploratory multigene and haplotype-based approaches focused on biologically interconnected pathways that may contribute jointly to AUD susceptibility.

Although large-scale GWAS have substantially advanced the understanding of the polygenic architecture of AUD, exploratory studies focused on biologically interconnected candidate genes and haplotypes may still provide complementary insights into pathway-level interactions, cohort-specific genetic patterns, and neurobiological mechanisms that are not fully captured by genome-wide approaches alone. Such analyses may also provide data that can be used in future meta-analyses or as hypothesis-generating evidence for subsequent pharmacogenetic studies, particularly if new therapeutic approaches targeting relevant biological pathways become available. Therefore, in this study, we aimed to explore the individual and combined contributions of selected polymorphisms in *GABRA1/2/6*, *BDNF*, and *DRD2*/*ANKK1* to AUD vulnerability, using a case–control genetic association design that compared individuals with AUD and healthy control participants.

The results of this exploratory study may provide additional insights into the potential role of these genetic variants and haplotypes in AUD susceptibility. Notably, this research is based on a Spanish cohort, addressing a key gap in the literature, as genetic studies on AUD within Spanish populations remain limited. Our approach highlights the need for further research on the genetic and neurobiological factors associated with AUD in geographically and clinically well-characterized European cohorts. In this context, exploratory multigene and haplotype-based analyses may help generate biologically informed hypotheses and improve the understanding of pathway-level mechanisms potentially involved in AUD susceptibility.

## 2. Results

### 2.1. GABRA Gene Polymorphisms

#### 2.1.1. GABRA1 Polymorphisms: rs1037715 and rs2279020

**Figure 1:**
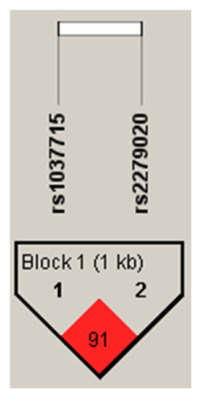
Linkage disequilibrium (LD) between rs1037715 and rs2279020 *GABRA1* polymorphisms. The numbers inside the squares represent |D’| × 100. Each cell is color-graded relative to the strength of LD between two markers. The absence of a value indicates complete linkage disequilibrium.

No significant differences were found when comparing the distribution of genotypes or allele frequencies for these polymorphisms between AUD patients and controls (Tables S1–S4). No deviations from the Hardy–Weinberg equilibrium (HWE) were detected in controls. Linkage disequilibrium (LD) was assessed using Haploview software for the analyzed polymorphisms (Figure 1). The corresponding logarithm of the odds (LOD) score was 30.68 (D’ = 0.91).

Using the expectation-maximization algorithm implemented in SHEsis, three haplotypes were identified (Table 1), without any significant association with AUD. No significant results were found when using Haploview for this purpose.

#### 2.1.2. GABRA2 Polymorphisms: rs71611977, rs279858, rs9291283, and rs894269

**Figure 2:**
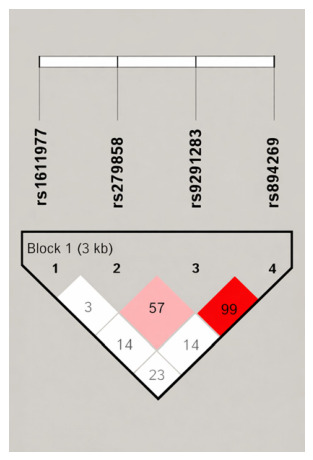
Linkage disequilibrium (LD) between the indicated *GABRA2* polymorphisms. The numbers inside the squares represent |D’| × 100. Each cell is color-graded relative to the strength of LD between two markers. The absence of a value indicates complete linkage disequilibrium.

No statistically significant differences were observed in the distribution of genotypes or allele frequencies for these polymorphisms between the AUD patients and controls (Tables S5–S11). No deviations from HWE were detected. For rs71611977, only AA and AG genotypes were detected, with no individuals carrying the GG genotype (all participants were AA or AG). To further explore the genetic architecture of the *GABRA2* gene, LD was assessed using Haploview for the analyzed polymorphisms (Figure 2). The corresponding LOD scores (Table 2) suggested strong LD between rs279858 and rs9291283 (D’ = 0.58, LOD = 13.16), and between rs9291283 and rs894269 (D’ = −0.99, LOD = 6.21).

The six most frequent haplotypes were identified using SHEsis (Table 3). No haplotype showed a significant association with AUD in this analysis, and these results were concordant with the findings obtained using Haploview; therefore, no statistically significant associations were found in the haplotype analysis of *GABRA2*.

#### 2.1.3. GABRA6 Polymorphisms: rs1992647, rs2197414, and rs3219151

No statistically significant differences were observed in genotype or allele distributions for rs2197414 and rs1992647 between AUD patients and controls (Tables S12–S15). No deviations from HWE were detected, and the observed allele frequencies were consistent with previously published data [37,38].

For rs3219151, non-significant differences in genotypic distribution were observed between AUD patients and controls (Table 4). Carriers of the T allele (CT + TT) were more frequent in AUD than in controls (79.8% vs. 72.2%; OR = 1.62; 95% CI = 1.02–2.59; *p* = 0.04). Likewise, the T-allele frequency was higher in AUD than in controls (57.9% vs. 49.0%; OR = 1.41; 95% CI = 1.03–1.93; *p* = 0.03) (Table 4).

Logistic regression analysis of genotypes under different inheritance models for the T allele (Table 5) showed a statistically significant difference between groups under the general model. Subjects with the TT genotype had 1.87-fold higher odds of developing AUD compared with the reference CC genotype (95% CI 1.02 to 3.42; *p* = 0.042). However, this association did not remain statistically significant after Bonferroni correction for the three genetic models tested within the SNP (α = 0.05/3 = 0.017), nor after age-adjusted sensitivity analysis.

**Figure 3:**
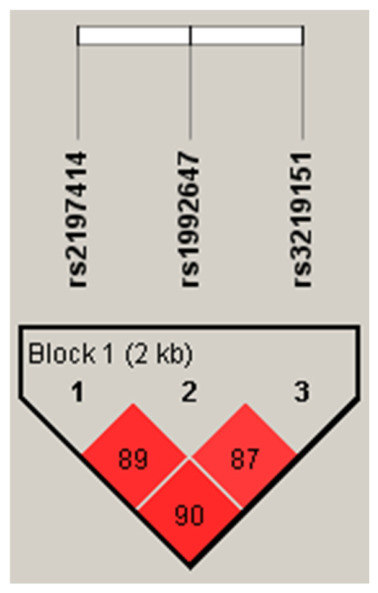
Linkage disequilibrium (LD) of the polymorphisms in the *GABRA6*.The numbers within the squares correspond to |D’| × 100. Each cell is color-graded relative to the strength of LD between two markers. The absence of value indicates complete linkage disequilibrium.

To further explore the genetic architecture of *GABRA6*, LD among the analyzed polymorphisms was assessed in Haploview (Figure 3). The corresponding D′ values indicated strong LD between– rs2197414 and rs1992647 (D′ = 0.898), between rs2197414 and rs3219151 (D′ = 0.906), and between rs1992647 and rs3219151 (D′ = 0.872). The associated LOD scores were high across pairs (rs2197414–rs1992647 LOD = 91.07; rs2197414–rs3219151 LOD = 62.19; rs1992647–rs3219151 LOD = 50.98) (Table 6).

Using the EM algorithm implemented in SHEsis, three common haplotypes were identified (Table 7). No risk-increasing haplotype reached statistical significance; however, the GAC haplotype (order rs2197414, rs1992647, rs3219151) was less frequent in AUD than in controls (0.071 vs. 0.122) and showed a protective association (OR = 0.58; 95% CI = 0.339–0.992; *p* = 0.045). This haplotype was also less frequent in AUD patients after analysis with Haploview (*p* = 0.029). The two remaining common haplotypes (CGC and GAT) did not reach statistical significance (Table 7).

### 2.2. Dopamine Receptor Polymorphisms: ANKK1/DRD2 (rs6277, rs1799978, and rs1800497)

Genotypic and allelic frequencies for rs6277, rs1799978, and rs1800497 did not differ significantly between AUD patients and controls. Genotype distributions in controls conformed to HWE (Tables S16–S20).

**Figure 4:**
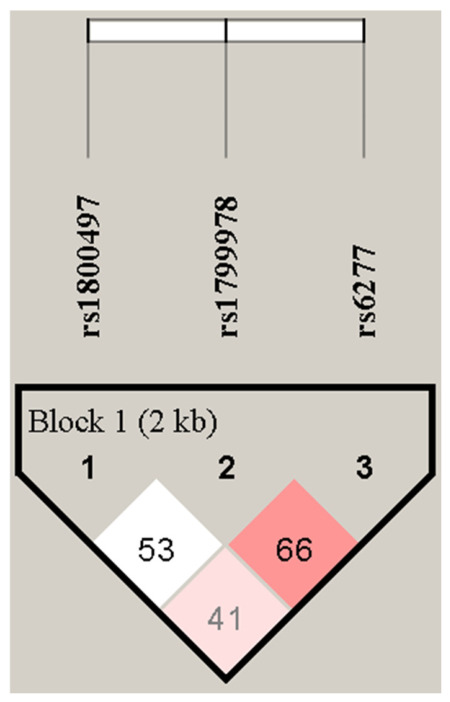
Linkage disequilibrium of the polymorphisms in the *DRD2* and *ANKK1* genes. The numbers inside the squares correspond to |D’| × 100. Each cell is color-graded relative to the strength of LD between two markers. The absence of a value indicates complete linkage disequilibrium.

In addition, LD among these variants was evaluated in Haploview (Figure 4). D′ values indicated modest-to-moderate LD (rs1800497–rs1799978 D′ = −0.532; rs1800497–rs6277 D′ = 0.416; rs1799978–rs6277 D′ = 0.663), with corresponding LOD scores providing evidence of LD for pairs involving rs6277 (rs1800497–rs6277 LOD = 4.39; rs1799978–rs6277 LOD = 2.39) and weaker support for rs1800497–rs1799978 (LOD = 0.29) (Table 8).

Haplotype analysis using SHEsis for the three-marker block (order rs1800497, rs1799978, rs6277) identified the five most frequent haplotypes (CTT, CTC, TTC, TTT, and CCC), but none showed a significant association with AUD (Table 9). Haplotype analysis using Haploview did not show any significant findings either. Overall, our data do not support a role for these *DRD2*/*ANKK1* variants in AUD susceptibility in our cohort.

### 2.3. BDNF Polymorphism (rs6265)

No statistically significant differences were observed in genotype or allele distributions of rs6265 between AUD patients and controls (Table 10). No deviations from HWE were detected, and the observed allele frequencies were consistent with previously published data.

No statistically significant differences were observed in allele distribution. The G allele appeared to be more frequent among AUD patients. Although not statistically significant, a lower proportion of A-allele carriers was observed in AUD patients compared with controls (38.5% vs. 47.8%; *p* = 0.083), along with a higher frequency of the A allele in controls than in patients (25.8% vs. 21.4%) (Table 10).

Logistic regression for rs6265 did not yield statistically significant effects at the prespecified threshold, but estimates were directionally consistent with a protective role of the A allele (Table 11). Age-adjusted sensitivity analysis did not yield a significant result either. In the general model, GA vs. GG showed OR = 0.66 (95% CI = 0.42–1.03; *p* = 0.067). In the dominant model (GA + AA vs. GG), OR = 0.68 (95% CI = 0.45–1.05; *p* = 0.084). The recessive model (AA vs. GA + GG) was non-significant (OR = 1.12; 95% CI = 0.38–3.31; *p* = 0.831).

Collectively, our results highlight the complex interplay between neuroplasticity, inhibitory neurotransmission, and dopaminergic signaling in AUD. In our sample, a specific *GABRA6* haplotype was associated with potential protective effects. Findings for BDNF suggested non-significant trends toward a potential protective effect. These findings support the potential utility of multigenic and haplotypic approaches for improving the understanding of biological mechanisms underlying AUD susceptibility. The main findings are summarized in Table 12.

## 3. Discussion

This study provides a comprehensive analysis of genetic variants in neuroplasticity, GABAergic, and dopaminergic systems in a well-characterized Spanish cohort, suggesting that specific polymorphisms and haplotypes may be associated with AUD.

Alcohol is known to have widespread effects throughout the brain, acting on diverse targets, including neurotransmitter membrane receptors. Therefore, it has been suggested that genetic vulnerability to alcoholism is likely due to a set of genes with small to moderate effects on neurotransmitter systems and signal transduction pathways [12,18,20,39].

GABA neurotransmitter receptors are key mediators of alcohol sedative and reinforcing effects and are considered to mediate several behavioral effects of this substance. As such, genes encoding GABA-related proteins have been proposed as functional candidate genes influencing the risk of AUD in different populations of European descent [40]. Among genes involved in the GABAergic system, particular attention has been given to *GABRA2* and *GABRA6*. Variants in *GABRA2* have been associated with differences in receptor sensitivity, altered inhibitory signaling, and modulation of reward pathways involving the mesolimbic system, as well as with individual differences in anxiety and impulsivity,—behavioral domains that are frequently associated with alcohol use disorder—and may also contribute to individual differences in susceptibility to alcohol-induced cirrhosis [32].

In our study, polymorphisms in several *GABRA* genes were examined. For *GABRA1*, no significant differences in genotype or allele frequency distributions were observed between AUD patients and controls. However, linkage disequilibrium analysis showed a strong association among the analyzed markers, suggesting that these polymorphisms are highly correlated and may be relevant for haplotype-based analyses. Similarly, four polymorphisms of *GABRA2* were studied (rs71611977, rs279858, rs9291283, and rs894269). However, individual SNPs showed no significant differences between AUD patients and controls. As previously described, rs71611977 presented only AA and AG genotypes, with no GG homozygotes observed [41]. No association was observed at the single variant level, and haplotype analyses did not reveal any significant association either. Previous studies have identified protective haplotypes in the *GABRA2* gene region [42,43], underscoring the importance of this genetic locus in AUD susceptibility and highlighting the value of haplotype-based analyses to detect genetic contributions that single-SNP approaches may overlook. However, no significant GABRA2 haplotype associations were detected in our Spanish cohort.

With respect to *GABRA6*, the analysis focused on polymorphisms rs1992647, rs2197414, and rs3219151. No significant differences were observed for rs1992647 and rs2197414 between AUD patients and controls, with allele frequencies in HWE and consistent with previous European reports [37,38,44]. In contrast, for rs3219151, although genotype-level differences did not reach strict significance, a suggestive pattern emerged: AUD patients showed a higher frequency of the T allele compared to controls. Allele-level comparisons showed a nominally higher frequency of the T allele in patients; however, this difference did not remain statistically significant after adjustment for multiple testing in logistic regression analyses.

Haplotype analyses revealed that the GAC haplotype (corresponding to rs1992647, rs2197414, and rs3219151 in order) was significantly more frequent in controls than in AUD patients, suggesting potential protective effects. To our knowledge, the putative protective effect of this haplotype in AUD has not been previously reported and is concordant with the potential protective effect of the C allele of rs3219151, observed in the present cohort. In addition, the GAT haplotype, containing the T allele of rs3219151, was more frequent in AUD patients, although it did not reach statistical significance.

While the precise functional effects of rs3219151 are not fully understood, the *GABRA6* rs3219151 T allele is associated with an increased stress response and a higher risk for current anxiety, depression, and suicidal ideation when individuals are exposed to significant recent negative life events [45]. Importantly, the T allele is in the 3′ untranslated region (3′UTR) of *GABRA6*, where it modulates gene expression by affecting mRNA stability and translation, which may impair GABA receptor function and enhance stress responses. This region interacts with microRNAs and RNA-binding proteins, leading to context-dependent changes in gene expression [45]. Our findings are consistent with previous associations of this SNP with AUD in studies of Taiwanese Han, Korean, Finnish, and Southwestern Native American populations [46] and with a prior report on the frequency of the rs3219151 T allele in European populations [47]. However, given the nominal significance levels observed and the exploratory design of the study, these findings should be interpreted with caution.

In this study, both European controls and AUD patients exhibited a higher T-allele frequency compared to Asian populations [47], underscoring the importance of population context in genetic association analyses. The higher baseline T-allele frequency in European cohorts suggests that the same variant’s effect size or detectability may differ by ancestry. Our results extend these observations to a Spanish population and suggest that rs3219151 may warrant further investigation as a potential contributor to AUD susceptibility, particularly in the context of GABAergic haplotypic variation. Notably, the T allele could increase the risk of alcohol-related disorders in individuals under high environmental stress [46]. However, replication in larger Spanish cohorts is needed to clarify the role of rs3219151 and to further support its contribution to AUD vulnerability in this population.

Collectively, our data are consistent with the possibility that rs3219151 may contribute modestly to AUD susceptibility, but *GABRA6* haplotypes capturing allelic interactions may better represent the functional impact on GABA_A receptor neurobiology, as evidenced by the protective GAC haplotype. Given that rs3219151 serves as a binding site for at least four microRNAs, it is plausible that variants at this locus modulate gene expression and receptor function by altering microRNA interactions [48,49]. Such mechanisms could contribute to the differences in AUD susceptibility observed in European vs. non-European populations [49,50]. In any case, our findings are consistent with a potential role of *GABRA6* in modulating inhibitory neurotransmission and the vulnerability to problematic alcohol use and provide data for further studies, including meta-analyses on this topic.

BDNF is a critical modulator of neuroplasticity and reward-related pathways that interact with both GABAergic and dopaminergic systems. In our Spanish cohort, the G (Val) allele of the *BDNF* rs6265 polymorphism was the most frequent among individuals with AUD, consistent with frequencies reported in other Caucasian populations [25,51]. This allele has been associated with poorer executive performance, suggesting it may contribute to AUD vulnerability by impairing cognitive control [51].

Similarly, we observed a higher frequency of the A allele (Met variant) in controls, while the G allele predominated in AUD patients; however, this finding did not reach statistical significance in our sample. Logistic regression analyses also showed non-significantly directionally lower odds estimates for the A allele. Consistent with our results, a recent study examining rs6265 in women with AUD reported a significant interaction between this polymorphism and personality traits. In that study, alcohol-dependent women with the GG genotype scored lower on agreeableness, whereas those with the GA genotype had higher anxiety levels, in a personality inventory. These findings suggest that certain personality factors may interact with *BDNF* genotype, potentially influencing the development of AUD in genetically susceptible individuals [34].

This study offers several notable contributions. Our research is based on European (especially Spanish) patients, addressing a gap in the literature, as many genetic studies of AUD have focused on non-European populations or on broad cohorts of European descent outside of specific European subpopulations. Additionally, by examining multiple genes and haplotypes simultaneously, our study provides a broader perspective than many previous studies, which tended to assess only one or two genes. Notably, the larger multigene studies of AUD have typically been conducted in non-European populations. By focusing specifically on male patients, we provide a more homogeneous analysis of genetic associations. Taken together, these aspects underscore the novelty and relevance of our work in advancing the understanding of AUD genetics in European populations. However, the present cohort was not designed to be population-representative, and the findings should therefore be interpreted as exploratory and hypothesis-generating. This is particularly relevant given that the heritability of AUD is estimated at around 50% [52], and that the prevalence of alcohol consumption in Spain is high [53].

However, we also acknowledge the limitations of the present study. The relatively small sample size and restriction to male participants may limit the generalizability of our findings. In particular, our all-male sample may not allow extrapolation of results to female AUD patients, in whom genetic and neurobiological mechanisms may differ. The inclusion of male participants reflects the availability of eligible patients during the recruitment period. Although this sex-homogeneous design reduces biological variability and increases precision for characterizing a particularly severe, high-risk alcohol-use phenotype that is more prevalent among men in many clinical settings, future studies including both sexes will therefore be important to assess the broader applicability of these findings and to determine whether the genetic associations and haplotypic patterns observed in this study differ according to sex-specific mechanisms involved in AUD susceptibility.

Additionally, the sample size was determined by the availability of a well-characterized clinical cohort of AUD patients and controls recruited over several years in a hospital-based setting, rather than by a predefined population-based sampling strategy. As is common in genetic association studies with strict clinical diagnostic criteria, no a priori statistical power calculation was performed. Consequently, the present study should be regarded as exploratory: although the available sample size may be sufficient to detect moderate-to-large effect sizes, there is a risk of false positives related to multiple comparisons, and smaller genetic effects may have remained undetected, particularly given the highly polygenic architecture of AUD.

Therefore, the absence of statistically significant associations for several analyzed variants, including those in *GABRA1*, *GABRA2*, *DRD2*/*ANKK1*, and BDNF, should not be interpreted as evidence of no effect or no association. Our post hoc power analysis indicated that the study had adequate power only to detect relatively large effects, whereas most AUD susceptibility loci identified in large-scale GWASs have smaller effect sizes. Thus, these negative findings may reflect limited statistical power rather than the true absence of a genetic contribution, and they should be interpreted cautiously. In addition, demographic differences between the groups, such as age, should be considered when interpreting the results. Therefore, the findings should be interpreted with caution and regarded as exploratory until confirmed in larger studies that can more comprehensively assess potential demographic and clinical confounding factors.

Although we applied a Bonferroni correction within each SNP to account for multiple inheritance models, no global correction across all analyzed genes and haplotypes was performed; therefore, some findings may be susceptible to type I error and should be interpreted with caution. This type of global adjustment is challenging in candidate-gene studies because the effective number of independent comparisons depends on the number of genes, loci, and haplotypic structures analyzed, as well as on linkage disequilibrium between variants. Nevertheless, we acknowledge that the observed associations were nominal and would not remain statistically significant under a strict global correction across all genetic markers and haplotypes. Therefore, these findings may be susceptible to type I error and should be interpreted with caution.

Furthermore, while the haplotype-based approach and the genetic homogeneity of the cohort may strengthen the robustness of our results, replication in independent cohorts is needed. Moreover, the lack of functional validation prevents direct conclusions about the underlying biological mechanisms.

Future studies incorporating functional assays, gene expression analyses, or experimental models, as well as replication in independent cohorts and diverse populations, will be essential to confirm the biological relevance of these variants and to elucidate their role in AUD susceptibility. This genetic vulnerability to AUD may arise from multiple molecular mechanisms, including the regulation of gene expression, alterations in protein structure and trafficking, and modulation of neurotransmitter receptor function. Overall, this study provides novel exploratory insights into potential genetic and haplotypic factors associated with AUD in a geographically homogeneous European cohort.

## 4. Materials and Methods

### 4.1. Patients and Controls

The study followed a case–control genetic association design and included 187 male AUD patients referred to the Alcoholism Unit of the University Hospital of Salamanca, Spain, and 160 healthy, sex-matched controls. Clinical and sociodemographic characteristics of the study population are summarized in Table S21. Only male participants were included due to the availability of eligible patients during the recruitment period.

AUD patients who had consumed more than 100 g of ethanol daily for at least 10 years. Participants were initially recruited using the Diagnostic and Statistical Manual of Mental Disorders, Fourth Edition (DSM-IV) criteria for alcohol dependence [54], but diagnostic concordance was optimal when the DSM-5 AUD criteria [55] were retroactively applied to these DSM-IV diagnosed cases, consistent with prior studies reporting strong agreement between DSM-IV dependence and moderate-to-severe DSM-5 AUD [56]. Given the clinical profile of the cohort, no distinction was made between moderate and heavy AUD. The mean age was 52.2 years (SD = 12.46) in the AUD group. Diagnoses were established through standard clinical psychiatric interviews conducted by a board-certified psychiatrist specialized in the treatment of alcoholism. Patients with addiction to substances other than nicotine or with major Axis I psychiatric disorders, such as mood disorders, schizophrenia, or significant anxiety disorders, were excluded. No formal structured diagnostic instrument was administered. The control group had a mean age of 46.6 years (SD = 19.56), consumed less than 10 g of ethanol per day and had no personal or family history (up to second degree) of alcohol abuse or dependence according to DSM-IV criteria (Table S21). Some participants from the present study cohort have been previously included in candidate-gene association studies investigating susceptibility to alcohol-related disorders [9,10,11].

All participants were Caucasian born in Castile and León (northwestern Spain), as were their parents and grandparents, ensuring population genetic homogeneity. All participants provided written informed consent, and the study protocol was approved by the University Hospital of Salamanca Ethics Committee.

### 4.2. Genotyping

Genomic DNA was extracted from nucleated peripheral blood cells by proteinase K digestion, followed by phenol-chloroform extraction and ethanol precipitation. DNA quality and concentration were assessed spectrophotometrically to ensure the integrity and purity of the samples, which were stored at −20 °C until processing. A total of 344 subjects, including AUD patients and healthy controls, were genotyped in random order on PCR plates. Selected polymorphisms in genes relevant to the neurobiology of addiction were studied: *GABRA1* (rs1037715 and rs2279020), *GABRA2* (rs71611977, rs279858, rs9291283, rs894269), *GABRA6* (rs1992647, rs2197414, rs3219151), *DRD2*/*ANKK1* (rs1800497, rs6277, rs1799978), and *BDNF* (rs6265). The effective sample size for each SNP (number of samples with valid genotype calls after quality control) is detailed in Table S22, which also includes genotyping call rates ranging from 90.5% to 99.4% across the analyzed SNPs. The selection of SNPs considered minor allele frequencies (MAFs) reported in international population databases, including the Ensembl genome browser (GRCh38) (https://www.ensembl.org) [57], based on the 1000 Genomes Project (Phase 3) data. Only polymorphisms with sufficient variability (MAF ≥ 5%) were generally included to improve power and avoid sparse genotype counts in our Spanish cohort. The corresponding IBS (Iberian populations in Spain) MAFs are detailed in Table S23, supporting the rationale that the selected polymorphisms are sufficiently variable in the reference population used to justify their analysis prior to linkage disequilibrium assessment. Variants with lower MAF values (e.g., rs1799978) were retained due to their potential functional relevance and prior evidence suggesting involvement in GABAergic or dopaminergic regulation. The selected SNPs were prioritized over other variants within the same genes based on:(i) prior evidence of association with alcohol use disorder or related neuropsychiatric phenotypes, (ii) their location in potentially functional regions (e.g., coding regions, untranslated regions, or regulatory loci), and (iii) their representativeness within haplotype blocks relevant for linkage disequilibrium analyses. Chromosomal locations, functional consequences, and population allele frequencies of all analyzed SNPs were obtained from the Ensembl genome browser (GRCh38) [57]. Major and minor alleles were assigned using the IBS population reference frequencies rather than the study sample to ensure independent classification. For variants for which IBS data were not accessible, allele frequencies were obtained from the gnomAD genomes dataset (Non-Finnish European population). For multiallelic variants, only alleles observed in our cohort were included in the association analyses. For rs6265 (BDNF Val66Met), alleles are annotated as C/T according to GRCh38; this corresponds to the G/A nomenclature commonly used in the clinical literature due to strand orientation (Table S23). Genotyping was performed using polymerase chain reaction (PCR) followed by a TaqMan assay (Applied Biosystems, Foster City, CA, USA) for allelic discrimination, ensuring high specificity and reproducibility of the results. VIC- and FAM probes were used for each polymorphism, with sequences detailed in Table S24. Amplification reactions were conducted on a StepOnePlus^®^ instrument (Applied Biosystems^®^), which allows multiple fluorescent channels for simultaneous detection of different genes.

PCR runs were carried out in a total volume of 10 μL, using TaqMan^®^ Universal PCR Master Mix No AmpErase^®^ UNG (Thermo Fisher Scientific, Applied Biosystems, Foster City, CA, USA), a specific probe for the polymorphism studied, sterile water, and genomic DNA.

### 4.3. Statistical Analysis

Differences in allele and genotype frequencies between groups were compared using the chi-square (χ^2^) test and, when necessary (expected values < 5), Fisher’s exact test. Deviation from the Hardy–Weinberg equilibrium in healthy controls was also assessed using the χ^2^ test. A *p*-value < 0.05 was considered statistically significant. Associations between each polymorphism and AUD were evaluated using logistic regression. For each SNP, we fitted separate logistic regression models assuming general, dominant, and recessive inheritance of the risk allele, with AUD status (patient vs. control) as the dependent variable and genotype as the main independent variable. Logistic regression analyses were performed without adjustment for age or other covariates, as the study was designed as an exploratory genetic association analysis focused on genotype differences within a geographically homogeneous population.

Odds ratios (OR) and 95% confidence intervals (CI) were estimated using the common homozygous genotype as the reference category. All statistical analyses were performed using SPSS version 15.0 (SPSS Inc., Chicago, IL, USA).

Haplotype frequencies, haplotypic odds ratios (OR) with 95% confidence intervals (95% CI), and pairwise linkage disequilibrium (LD) were estimated to evaluate genetic associations. Haplotype frequencies were inferred using the expectation-maximization algorithm implemented in SHEsis (online version, available at: http://analysis.bio-x.cn, accessed on 21 November 2025), and case–control associations were tested by chi-square test and permutation [58,59]. To confirm the accuracy of the haplotype estimations and to visualize LD patterns, additional analyses were performed using Haploview 4.2 [60], which also provided graphical representations of LD patterns (D’ values) and corresponding logarithm of the odds (LOD) scores (available at: http://www.broadinstitute.org/haploview, accessed on 22 November 2025 ). All haplotype analyses were cross-validated using both programs to ensure consistency of results.

Multiple testing was addressed at the level of individual SNP analyses. When multiple inheritance models (general, dominant, and recessive) were tested for the same SNP, a Bonferroni correction was applied for the number of models tested, resulting in a corrected significance threshold of α = 0.05/3. Given the exploratory design and candidate-gene approach, no additional correction was applied across different genes or haplotypes.

A post hoc power calculation was performed for the case–control genetic association analysis assuming an allelic effect and using a two-sample test for differences in proportions at the individual level, which is equivalent to a 1-degree-of-freedom chi-square allelic association test under Hardy–Weinberg equilibrium. The calculation was based on 187 cases and 160 controls, an α level of 0.05, and a minor allele frequency of 0.20 in controls. Under these assumptions, the study had a statistical power of 80.5% to detect an allelic odds ratio of 2.0 for the development of alcohol use disorder.

## 5. Conclusions

Our study provides exploratory insights into potential genetic factors associated with AUD in a geographically homogeneous Spanish cohort. The potential value of these findings lies primarily in their ability to inform future research on the biological heterogeneity of AUD and to contribute data for future integrative analyses, rather than in immediate clinical translation.

The results suggest a possible protective association of the GABRA6 rs3219151 C allele in this Spanish cohort, while emphasizing the value of haplotype-based approaches to reveal allelic interactions that may be relevant to neurobiological pathways related to GABAergic signaling, dopaminergic function, and neuroplasticity. These findings reinforce the complex interplay between inhibitory neurotransmission, the reward circuitry, and executive function in AUD susceptibility. Despite limitations related to sample size, the male-only sample, and the nominal statistical significance of the observed associations, this exploratory work highlights the importance of multigene analyses and provides a basis for future research and replication studies in larger and independent cohorts before definitive conclusions can be drawn. Overall, these results provide exploratory insights into the potential contribution of specific genetic variants and haplotypes to AUD susceptibility and may contribute to future studies exploring the complex genetic and neurobiological mechanisms underlying AUD.

## Figures and Tables

**Table 1:** Haplotype analysis results for *GABRA1* polymorphisms comparing AUD patients versus controls.

Haplotype	Frequency (AUD Patients)	Frequency (Controls)	Haplotype OR (95% CI)	*p*
CA	0.199	0.233	0.842 (0.581~1.219)	0.363
CG	0.606	0.573	1.191 (0.881~1.61)	0.254
TG	0.182	0.19	0.976 (0.66~1.445)	0.907

Haplotypes are constructed in the order rs1037715–rs2279020. Major alleles in the Iberian populations in Spain (IBS) are C (rs1037715) and A (rs2279020).

**Table 2:** Linkage disequilibrium of polymorphisms located in the *GABRA2* gene.

	rs71611977	rs279858	rs9291283	rs894269
rs71611977		0.036	−0.144	0.234
rs279858	0		0.580	0.143
rs9291283	0.02	13.16		−0.99
rs894269	0.59	0.5	6.21	

D′ values are shown above the diagonal; the corresponding LOD scores are shown below the diagonal.

**Table 3:** Haplotype analysis results for *GABRA2* polymorphisms comparing AUD patients versus controls.

Haplotype	Frequency (AUD Patients)	Frequency (Controls)	Haplotype OR (95% CI)	*p*
ACAC	0.041	0.059	0.748 (0.367~1.524)	0.423
ACGC	0.425	0.43	1.118 (0.824~1.518)	0.47
ATAC	0.208	0.185	1.272 (0.862~1.876)	0.224
ATGC	0.116	0.125	1.004 (0.625~1.61)	0.986
ACGT	0.072	0.094	0.815 (0.465~1.428)	0.475
ATGT	0.094	0.08	1.298 (0.748~2.255)	0.351

The polymorphisms within each haplotype are presented in the following order: rs71611977, rs279858, rs9291283, and rs894269.

**Table 4:** Distribution of genotypes and alleles for the rs3219151 polymorphism in the *GABRA6* gene.

Group	Individuals	Genotypic Frequencies (%)			Allelic Presence		Allelic Frequencies (%)	
		CC	CT	TT	CC + CT	CT + TT	Allele C	Allele T
AUD	178	36 (20.20)	78 (43.80)	64 (36.00)	114 (64.00)	142 (79.80)	150 (42.10)	206 (57.90) ^b^
Controls	144	40 (27.8)	66 (45.8)	38 (26.4)	106 (73.60)	104 (72.20) ^a^	146 (50.70)	142 (49.3)

The table shows a comparative analysis between AUD patients and controls. Major allele: C. Minor allele: T, defined according to the Iberian population in Spain (IBS) allele frequencies. Genotypic distribution (*p* = 0.14). ^a^ T allele carriers (CT + TT): *p* = 0.04; OR = 1.62 (95% CI = 1.02–2.59). ^b^ T allele frequency in the AUD patient group vs. control (T vs. C): *p* = 0.03; OR = 1.41 (95% CI = 1.03–1.93).

**Table 5:** Logistic regression analysis of genotypes for the rs3219151 polymorphism in *GABRA6* according to different inheritance models of the T allele in AUD patients versus controls.

			Logistic Regression Analysis		
Model	AUD Patients (%)	Controls (%)	OR	95% CI	*p*
General					
CC	36 (20.22)	40 (27.78)	1	Reference	
CT	78 (43.82)	66 (45.83)	1.31	0.91–2.54	0.114
TT	64 (35.96)	38 (26.39)	1.87	1.02–3.42	0.042
Dominant					
CC	36 (20.22)	40 (27.78)	1	Reference	
CT + TT	142 (79.78)	104 (72.22)	1.52	0.91–2.54	0.114
Recessive					
CT + CC	114 (64.04)	106 (73.61)	1	Reference	
TT	64 (35.96)	38 (26.39)	1.57	0.97–2.53	0.067

Logistic regression analysis of rs3219151 GABRA6 genotypes under different inheritance models of the T allele in alcohol use disorder (AUD) patients versus controls. Major allele: C. Minor allele: T, as defined by Iberian population in Spain (IBS) frequencies. Values are OR with 95% CI and corresponding *p*-values. Comparisons were performed under the general (CC vs. CT vs. TT), dominant (CT + TT vs. CC), and recessive (TT vs. CC + CT) inheritance models. Reference categories: CC genotype in the general and dominant models, and CT plus CC genotypes in the recessive model.

**Table 6:** Linkage disequilibrium of polymorphisms located in the *GABRA6*.

	rs2197414	rs1992647	rs3219151
rs2197414		0.898	0.906
rs1992647	91.07		0.872
rs3219151	62.19	50.98	

D′ values are shown above the diagonal; the corresponding LOD scores are shown below the diagonal.

**Table 7:** Haplotype analysis results in AUD patients versus controls for *GABRA6* polymorphisms.

Haplotype	Frequency (AUD Patients)	Frequency (Controls)	Haplotype OR (95% CI)	*p*
CGC	0.295	0.367	0.774 (0.559~1.071)	0.121
GAT	0.54	0.482	1.319 (0.978~1.778)	0.069
GAC	0.071	0.122	0.58 (0.339~0.992)	0.045

The polymorphisms within each haplotype are presented in the order rs2197414, rs1992647, and rs3219151.

**Table 8:** Linkage disequilibrium of polymorphisms located in *DRD2* and *ANKK1*.

	rs1800497	rs1799978	rs6277
rs1800497		−0.532	0.416
rs1799978	0.29		0.663
rs6277	4.39	2.39	

D′ values are shown above the diagonal; the corresponding LOD scores are shown below the diagonal.

**Table 9:** Haplotype analysis results in AUD patients versus controls for *DRD2* and *ANKK1* polymorphisms.

Haplotype	Frequency (AUD Patients)	Frequency (Controls)	Haplotype OR (95% CI)	*p*
CTT	0.543	0.534	1.023 (0.787~1.33)	0.862
CTC	0.251	0.25	1.002 (0.73~1.376)	0.987
TTC	0.127	0.134	0.938 (0.62~1.419)	0.763
TTT	0.04	0.044	0.895 (0.445~1.8)	0.756
CCC	0.027	0.032	0.852 (0.372~1.949)	0.704

The polymorphisms within each haplotype are presented in the order rs1800497, rs1799978, and rs6277.

**Table 10:** Distribution of genotypes and alleles for the rs6265 polymorphism in the *BDNF* gene.

Group	Individuals	Genotypic Frequencies (%)			Allelic Presence (%)		Allelic Frequencies	
		GG	AG	AA	GG + GA	GA + AA	Allele G	Allele A
AUD	187	115 (61.5)	64 (34.2)	8 (4.3)	179 (95.7)	72 (38.50)	294 (78.61)	80 (21.39)
Controls	157	82 (52.2)	69 (43.9)	6 (3.8)	151 (96.20)	75 (47.80)	233 (74.20)	81 (25.80)

Major allele: G. Minor allele: A, according to Iberian population in Spain (IBS) frequencies. Genotypic and allelic frequencies are presented as numbers and percentages.

**Table 11:** Logistic regression analysis of rs6265 *BDNF* genotypes according to different inheritance models of the G allele in AUD patients versus controls.

			Logistic Regression Analysis		
Model	AUD Patients (%)	Controls (%)	OR	95% CI	*p*
General					
GG	115 (61.50)	82 (52.23)	1	Reference	
GA	64 (34.22)	69 (43.95)	0.66	0.42–1.03	0.067
AA	8 (4.28)	6 (3.82)	0.95	0.32–2.84	0.928
Dominant					
GG	115 (61.50)	82 (52.23)	1	Reference	
GA + AA	72 (38.50)	75 (47.77)	0.68	0.45–1.05	0.084
Recessive					
GG + GA	179 (95.72)	151 (96.18)	1	Reference	
AA	8 (4.28)	6 (3.82)	1.12	0.38–3.31	0.831

Logistic regression analysis of rs6265 *BDNF* genotypes under different inheritance models of the G allele in AUD patients vs. controls. Major allele: G. Minor allele: A, defined according to Iberian population in Spain (IBS) frequencies. Comparisons were performed under the general (GG vs. GA vs. AA), dominant (GG vs. GA + AA), and recessive (AA vs. GA + GG) inheritance models. Reference categories: GG genotype in the general and dominant models, and GA plus GG genotypes in the recessive model. Values are OR with 95% CI and corresponding *p*-values.

**Table 12:** Genetic variants of interest in AUD in a Spanish cohort.

Gene	SNP/Haplotype	Main Findings in AUD vs. Controls	OR (95% CI)	*p*- Value
*GABRA6*	rs3219151 (CT + TT vs. CC)	Higher frequency of T-allele carriers in AUD patients	1.62 (1.02–2.59)	0.04
	rs3219151 (T vs. C allele)	Higher T-allele frequency in AUD patients	1.41 (1.03–1.96)	0.03
	rs3219151 (TT vs. CC, general model)	Higher frequency of TT genotype in AUD patients	1.87 (1.02–3.42)	0.042 *
	GAC haplotype (rs2197414–rs1992647–rs3219151)	Lower frequency in AUD patients than in controls	0.58 (0.339–0.992)	0.045
*BDNF*	rs6265 (GA vs. GG)	Lower frequency of GA genotype in AUD patients	0.66 (0.42–1.03)	0.067
	rs6265 (GA + AA vs. GG)	Lower frequency of A- allele carriers in AUD patients	0.68 (0.45–1.05)	0.084

AUD: alcohol use disorder. * *p* = 0.042 did not meet statistical significance after Bonferroni correction.

## Data Availability

The data presented in the current study are available upon request from the corresponding author. Due to ethical considerations, the data are not publicly accessible.

## Supplementary Materials

The following supporting information can be downloaded at: https://www.mdpi.com/article/10.3390/ijms27125376/s1.
