# Supplementary material for: Exploratory Analysis of Genetic Variants in BDNF, GABA Receptors, and Dopaminergic Pathways with Alcohol Use Disorder in a Spanish Cohort
Source: Int J Mol Sci. 2026 Jun 15;27(12):5376. doi: 10.3390/ijms27125376 (PMC13299486; doi:10.3390/ijms27125376)
Supplement: Supplementary file 1 [file ijms-27-05376-s001.zip › ijms-4306130-supplementary.pdf]

Supplementary Material

1. *GABRA1* gene polymorphism

A. rs1037715 polymorphism

Table S1. Distribution of genotypes for the rs1037715 polymorphism of *GABRA1*

**Group**	Number of Individuals	Genotype Frequencies (%)		
		CC	CT	TT
AUD	180	120 (66.7%)	50 (27.8%)	10 (5.6%)
Control	150	101 (67.3%)	40 (26.7%)	9 (6.0%)

Hardy–Weinberg equilibrium: $\chi^2 = 3.156$, $p = 0.076$

Table S2. Distribution of alleles for the rs1037715 polymorphism of *GABRA1*

Group	Number of Individuals	Allelic Presence		Allelic Frequencies	
		CC + CT	*CT+TT*	*Allele C*	*Allele T*
AUD	180	170 (94.4%)	60 (33.3%)	290 (80.6%)	70 (19.4%)
Control	150	141 (94.0%)	49 (32.7%)	242 (80.7%)	58 (19.3%)

B. rs2279020 polymorphism

Table S3. Distribution of genotypes for the rs2279020 polymorphism of *GABRA1*

**Group**	Number of Individuals	Genotype Frequencies (%)		
		AA	*AG*	*GG*
AUD	184	67 (36.4%)	95 (51.6%)	22 (12.0%)
Control	160	56 (35.0%)	75 (46.9%)	29 (18.1%)

Hardy–Weinberg equilibrium: $\chi^2 = 0.37$, $p = 0.544$

Table S4. Distribution of alleles for the rs2279020 polymorphism of *GABRA1*

Group	Number of Individuals	Allelic Presence		Allelic Frequencies	
		AA + AG	*AG+GG*	*Allele A*	*Allele G*
AUD	184	162 (88.0%)	117 (63.6%)	229 (62.2%)	139 (37.8%)
Control	160	131 (81.9%)	104 (65.0%)	187 (58.4%)	133 (41.6%)

2. *GABRA2* polymorphism

A. rs279858 polymorphism

Table S5. Distribution of genotypes for the rs279858 polymorphism of *GABRA2*

**Group**	Number of Individuals	Genotype Frequencies (%)		
		CC	*CT*	*TT*
AUD	182	42 (23.1%)	80 (44.0%)	60 (33.0%)
Control	149	25 (16.8%)	68 (45.6%)	56 (37.6%)

Hardy–Weinberg equilibrium: $\chi^2 = 0.315$, $p = 0.575$

Table S6. Distribution of alleles for the rs279858 polymorphism of *GABRA2*

Group	Number of Individuals	Allelic Presence		Allelic Frequencies	
		CC + CT	*CT+ TT*	*Allele C*	*Allele T*
AUD	182	122 (67.0%)	140 (76.9%)	164 (45.1%)	200 (27.5%)
Control	149	93 (62.4%)	124 (83.2%)	118 (39.6%)	180 (60.4%)

B. rs71611977 polymorphism

Table S7. Distribution of genotypes and alleles for the rs71611977 polymorphism of *GABRA2*

Group	Number of Individuals	Genotype Frequencies (%)		Allelic Frequencies	
		AA	*AG*	*A*	*G*
AUD	183	168 (91.8%)	15 (8.2%)	351(95.9%)	15(4.1%)
Control	151	142 (94.0%)	9 (6.0%)	293(97.0%)	9(3.0%)

Hardy–Weinberg equilibrium: $\chi^2 = 0.142$, $p = 0.706$

C. rs9291283 polymorphism

Table S8. Distribution of genotypes for the rs9291283 polymorphism of *GABRA2*

**Group**	Number of Individuals	Genotype Frequencies (%)		
		AA	*AG*	*GG*
AUD	183	12 (6.6%)	74 (40.4%)	97 (53.0%)
Control	151	10 (6.6%)	55 (36.4%)	86 (57.0%)

Hardy–Weinberg equilibrium: $\chi^2 = 0.090$, $p = 0.765$

Table S9. Distribution of alleles for the rs9291283 polymorphism of *GABRA2*

Group	Number of Individuals	Allelic Presence		Allelic Frequencies	
		AA + AG	*AG+GG*	*Allele A*	*Allele G*
AUD	183	86 (47.0%)	171 (93.4%)	98 (26.8%)	268 (73.2%)
Control	151	65 (43.0%)	141 (93.4%)	75 (24.8%)	227 (75.2%)

D. rs894269 polymorphism

Table S10. Distribution of genotypes for the rs894269 polymorphism of *GABRA2*

Group	Number of Individuals	Genotype Frequencies (%)		
		CC	*CT*	*TT*
AUD	180	122 (67.8%)	51 (28.3%)	7 (3.9%)
Control	151	103 (68.2%)	39 (25.8%)	9 (6.0%)

Hardy–Weinberg equilibrium: $\chi^2 = 3.703$, $p = 0.054$

Table S11. Distribution of alleles for the rs894269 polymorphism of *GABRA2*

Group	Number of Individuals	Genotype Frequencies (%)		Allelic Frequencies	
		CC + CT	*CT+TT*	*Allele C*	*Allele T*
AUD	180	173 (96.1%)	58 (32.2%)	295 (81.9%)	65 (18.1%)
Control	151	142 (94.0%)	48 (31.8%)	245 (81.1%)	57 (18.9%)

3. *GABRA6* polymorphisms
A. rs2197414 polymorphism

Table S12. Distribution of genotypes for the rs2197414 polymorphism of *GABRA6*

Group	Number of Individuals	Genotype Frequencies (%)		
		CC	*CG*	*GG*
AUD	187	81 (43.3%)	79 (42.2%)	27 (14.4%)
Control	154	60 (39.0%)	66 (42.9%)	28 (18.2%)

Hardy–Weinberg equilibrium: $\chi^2 = 3.140$, $p = 0.535$

Table S13. Distribution of alleles for the rs2197414 polymorphism of *GABRA6*

Group	Number of Individuals	Allelic Presence		Allelic Frequencies	
		CC + GC	*GC+GG*	*Allele C*	*Allele G*
AUD	187	160 (85.6%)	106 (56.7%)	241 (64.4%)	133 (35.6%)
Control	154	126 (81.8%)	94 (61.0%)	186 (60.4%)	122 (39.6%)

B. rs1992647 polymorphism

Table S14. Distribution of genotypes for the rs1992647 polymorphism of *GABRA6*

Group	Number of Individuals	Genotype Frequencies (%)		
		AA	*AG*	*GG*
AUD	187	84 (44.9%)	77 (41.2%)	26 (13.9%)
Control	158	63 (3.9%)	69 (43.7%)	26 (16.5%)

Hardy–Weinberg equilibrium: $\chi^2 = 0.996$, $p = 0.318$

Table S15. Distribution of alleles for the rs1992647 polymorphism of *GABRA6*

Group	Number of Individuals	Allelic Presence		Allelic Frequencies	
		AA + AG	*AG+GG*	*Allele A*	*Allele G*
AUD	187	161 (86.1%)	103 (55.1%)	245 (65.5%)	129 (34.5%)
Control	158	132 (83.5%)	95 (60.1%)	195 (61.7%)	121 (38.3%)

4. Polymorphisms of the *DRD2* and *ANKK1* genes

A. rs6277 polymorphism

Table S16. Distribution of genotypes for the rs6277 polymorphism of *DRD2*

Group	Number of Individuals	Genotype Frequencies (%)		
		TT	*CT*	*CC*
AUD	181	63 (34.8%)	92 (50.8%)	26 (14.4%)
Control	149	48 (32.2%)	75 (50.3%)	26 (17.4%)

Hardy–Weinberg equilibrium: $\chi^2 = 0.390$, $p = 0.532$

Table S17. Distribution of alleles for the rs6277 polymorphism of *DRD2*

Group	Number of Individuals	Allelic Presence		Allelic Frequencies	
		TT+CT	*CT+CC*	*Allele T*	*Allele C*
AUD	181	155 (85.6%)	118 (65.2%)	218 (60.2%)	144 (39.8%)
Control	149	123 (82.6%)	101 (67.8%)	171 (57.4%)	127 (42.6 %)

B. rs1799978 polymorphism

Table S18. Distribution of genotypes for the rs1799978 polymorphism of *DRD2*

Group	Number of Individuals	Allelic Presence		Allelic Frequencies	
		TT	*TC*	*T*	*C*
AUD	168	155 (92.3%)	13 (7.7%)	323(96.1%)	13(3.9%)
Control	148	137 (92.6%)	11 (7.4%)	285(96.3%)	11(3.7%)

-Hardy–Weinberg equilibrium: $\chi^2 = 0.730$, P = 0.393
-No CC genotype was observed for rs1799978 in our sample

C. 1

Table S19. Distribution of genotypes for the rs1800497 polymorphism of *ANKK1*

Group	Number of Individuals	Genotype Frequencies (%)		
		CC	*CT*	*TT*
AUD	175	118 (67.4%)	54 (30.9%)	3 (1.7%)
Control	139	92 (66.2%)	40 (28.8%)	7 (5.0%)

Hardy–Weinberg equilibrium: $\chi^2 = 0.015$, $p = 0.901$

Table S20. Distribution of alleles for the rs1800497 polymorphism of *ANKK1*

Group	Number of Individuals	Allelic Presence		Allelic Frequencies	
		CC+CT	*CT+TT*	*Allele C*	*Allele T*
AUD	175	172 (98.3%)	57 (32.6%)	290 (82.9%)	60 (17.1%)
Control	139	132 (95.0%)	47 (33.8%)	224 (80.6%)	54 (19.4 %)

Table S21. Clinical and demographic characteristics of AUD patients and controls

**Variable**	**AUD patients (n=187)**	**Controls (n=160)**
Age, years	52.2 (12.46)	46.6 (19.5)
Male sex	187 (100%)	160 (100%)
Daily alcohol intake (g/day)	>100	<10

Values are presented as mean (standard deviation) for continuous variables and as number (percentage) for categorical variables. All AUD patients reported at least 10 years of excessive ethanol intake. AUD: alcohol use disorder.

Table S22. Effective sample size and genotyping call rates for each analyzed SNP

Gene	SNP	Cases Genotyped (n)	Controls Genotyped (n)	Total Genotyped (n)	Call Rate (%)
GABRA1	rs1037715	180	150	330	95.1%
	rs2279020	184	160	344	99.1%
GABRA2	rs279858	182	149	331	95.4%
	rs71611977	183	151	334	96.3%
	rs9291283	183	151	334	96.3%
	rs894269	180	151	331	95.4%
GABRA6	rs2197414	187	154	341	98.3%
	rs1992647	187	154	345	99.4%
	rs3219151	178	144	322	92.8%
DRD2 and *ANKK1*	rs6277	181	149	330	95.1%
	rs1799978	168	148	316	91.1%
	rs1800497	175	139	314	90.5%
BDNF	rs6265	187	157	344	99.1%

Genotyped (n) indicates the number of samples with valid genotype calls after quality control

Table S23. Genomic characteristics and population allele frequencies of the analyzed SNPs (GRCh38 assembly; 1000 Genomes IBS, with gnomAD used where IBS data were unavailable)

Gene	SNP	Functional consequence	Major allele	Minor allele	MAF (IBS or gnomAD)	Chromosome location
GABRA1	rs1037715	Intron	C	T	0.136	5:161888763
	rs2279020	Intron	A	G	0.304	5:161895883
GABRA2	rs279858	Missense	T	C	0.425	4:46312576
	rs9291283	Intron	G	A	0.294	4:46369816
	rs894269	Intron	C	A<br>T	0.070<br>0.112	4:46391595
	rs71611977	Intron	A	G	0.03	4:46351593
GABRA6	*rs2197414	Intergenic	C	G	0.367	5:161681878
	rs1992647	Intergenic	A	G	0.336	5:161684168
	rs3219151	3'UTR	T	C	0.393	5:161701908
DRD2 and ANKK1	rs6277	3'UTR	A	G	0.421	11:113412737
	rs1799978	Regulatory region	T	C	0.056	11:113475629
	rs1800497	Missense	G	A	0.145	11:113400106
BDNF	rs6265	Missense	C	T	0.210	11:27658369

Major and minor alleles were defined according to allele frequencies in the Iberian populations in Spain (IBS) as reported in the Ensembl genome browser (GRCh38) (https://www.ensembl.org) based on the 1000 Genomes Project (Phase 3) data. Minor allele frequency (MAF) refers to the frequency of the less common allele in the IBS population. For variants marked with (*), for which IBS data were not accessible, allele frequencies were obtained from the gnomAD

genomes dataset (Non-Finnish European population). Functional consequences correspond to Ensembl (GRCh38). rs894269 is reported as a multiallelic variant in the IBS population, with minor alleles T (0.112) and A (0.070). However, only the C and T alleles were observed in our cohort and therefore only these alleles were included in the association analyses. Chromosomal locations correspond to the GRCh38 assembly. For rs6265, C/T genomic annotation corresponds to the G/A (Val66Met) clinical nomenclature due to strand orientation.

Table S24. VIC and FAM probe sequences used for genotyping polymorphisms in *GABRA1*, *GABRA2*, *GABRA6*, *DRD2/ANKK1*, and B*DNF* genes

Gene	Polymorphism	Reference	VIC and FAN probes
GABRA1	rs1037715	C_1667770_10	**VIC:** CATTTTTCTTAAATTTGGATCACTT[C]CTTCTTTTCAAATGACCTTAAAGAT<br>**FAM:** CATTTTTCTTAAATTTGGATCACTT[T]CTTCTTTTCAAATGACCTTAAAGAT
	rs2279020	C_15966883_10	**VIC:** TTCCAGAAAAGGTAAATGCTTTAAT[A]GTCACTGTAGTACATCAATATTATG<br>**FAM:** TTCCAGAAAAGGTAAATGCTTTAAT[G]GTCACTGTAGTACATCAATATTATG
GABRA2	rs71611977	C__96990332_10	**VIC:** TAGAGAGACAAGAAATATATTTTCT[A]CAGACAAAATTTTTAAAAGATATAT<br>**FAM:** TAGAGAGACAAGAAATATATTTTCT[G]CAGACAAAATTTTTAAAAGATATAT
	rs279858	C_2073557_10	**VIC:** TTGTCATATTATGAGCTACTGATTT[C]TTCCCATTGTGAAAAAAGGTATCTG<br>**FAM:** TTGTCATATTATGAGCTACTGATTT[T]TTCCCATTGTGAAAAAAGGTATCTG
	rs9291283	C_8262290_10	**VIC:** TTAGAGTTATATTAAATATAGTAAC[A]GGAGATTTGTTCCAAAGGAAATGTG<br>**FAM:** TTAGAGTTATATTAAATATAGTAAC[G]GGAGATTTGTTCCAAAGGAAATGTG
	rs894269	C_8263129_10	**VIC:** ATATGTTACAACCTAAACAAACATT[C]GCTCTCTTATTTAGCTTGCTCTGTG<br>**FAM:** ATATGTTACAACCTAAACAAACATT[T]GCTCTCTTATTTAGCTTGCTCTGTG
GABRA 6	rs1992647	C_11275607_10	**VIC:** CTCTGATGACACAAATCAGAGATGA[A]TGGTAAACATTTCGATAGGAAAAAA<br>**FAM:** CTCTGATGACACAAATCAGAGATGA[G]TGGTAAACATTTCGATAGGAAAAAA
	rs2197414	C_1703387_10	**VIC:** CGAGGAACATTTTACTAAAGTTTGA[C]TGGTTTTATTCTAGAGCAAGTTTTC<br>**FAM:** CGAGGAACATTTTACTAAAGTTTGA[G]TGGTTTTATTCTAGAGCAAGTTTTC
	rs3219151	C_11263956_10	**VIC:** AATTGGAAATCTGTAACGCAGCTTC[C]GTAAGCATGTGTGGGCAAAAAAGCA<br>**FAM:** AATTGGAAATCTGTAACGCAGCTTC[T]GTAAGCATGTGTGGGCAAAAAAGCA
DRD2 /ANKK1	rs6277	C_11339240_10	**VIC:** TCTTCTCTGGTTTGGCGGGGCTGTC[A]GGAGTGCTGTGGAGACCATGGTGGG<br>**FAM:** TCTTCTCTGGTTTGGCGGGGCTGTC[G]GGAGTGCTGTGGAGACCATGGTGGG
	rs1799978	C_7486599_20	**VIC:** GCGCTCCCACCCACACCCAGAGTAA[C]AAGCTGTGATTGCAGGCTGGGTCCT<br>**FAM:** GCGCTCCCACCCACACCCAGAGTAA[T]AAGCTGTGATTGCAGGCTGGGTCCT
	rs1800497	C_7486676_10	**VIC:** CACAGCCATCCTCAAAGTGCTGGTC[A]AGGCAGGCGCCCAGCTGGACGTCCA<br>**FAM:** CACAGCCATCCTCAAAGTGCTGGTC[G]AGGCAGGCGCCCAGCTGGACGTCCA

BDNF	rs6265	C_11592758_10	**VIC:** TCCTCATCCAACAGCTCTTCTATCA[C]GTGTTCGAAAGTGTCAGCCAATGAT<br>**FAM:** TCCTCATCCAACAGCTCTTCTATCA[T]GTGTTCGAAAGTGTCAGCCAATGAT
